# The Efficacy of Pelvic Floor Rehabilitation in the Treatment of Urinary Incontinence in Female Athletes: A Systematic Review

**DOI:** 10.3390/sports12120338

**Published:** 2024-12-05

**Authors:** Andrea Demeco, Giulia Bartocci, Noemi Astore, Beatrice Vignali, Antonello Salerno, Stefano Palermi, Ruben Foresti, Chiara Martini, Cosimo Costantino

**Affiliations:** 1 Department of Medicine and Surgery, University of Parma, 43126 Parma, Italy; giulia.bartocci@unipr.it (G.B.); noemi.astore@unipr.it (N.A.); beatrice.vignali@unipr.it (B.V.); antonello.salerno@unipr.it (A.S.); ruben.foresti@unipr.it (R.F.); chiara.martini@unipr.it (C.M.); cosimo.costantino@unipr.it (C.C.); 2 Public Health Department, University of Naples Federico II, 80131 Naples, Italy; stefano.palermi@unina.it

**Keywords:** pelvic floor, pelvic rehabilitation, sports, pelvic management

## Abstract

Background and Objectives: Urinary incontinence (UI) prevalence reaches the 80% rate in female athletes involved in high-impact sports. In this context, although conservative treatment represents the first therapeutic choice, there is still a lack of knowledge on the efficacy of conservative programs in young female athletes. Therefore, the aim of this study was to investigate the role of pelvic floor rehabilitation in the treatment of UI in young nulliparous female athletes. We performed a literature search using PubMed, Medline, Cochrane Library, Web of Science, and Scopus. The selection of articles was conducted using a specific search string: “[((pelvic floor dysfunction) OR (urinary incontinence) OR (dyspareunia) OR (dysuria)) AND ((sport) OR (sports)) AND ((female) OR (woman) OR (women) OR (girl)) AND ((rehabilitation) OR (rehab) OR (pelvic rehabilitation) OR (exercise))]”. The review protocol was registered in PROSPERO with the ID CRD42024559990. A total of 1018 articles were found in all searches of the databases. After removing duplicates, 663 papers were reviewed in terms of title and abstract. Finally, a total of six studies were included in the present review. The results of this review show that conservative treatment with a personalised pelvic floor muscle training program (PFMTP) represents an effective treatment for UI, decreasing urine loss and improving maximum voluntary pelvic contraction; this is linked with an improvement in quality of life and sports performance, in particular when supervised by a physical therapist. Moreover, due to the reluctance of athletes to talk about UI, an educational program should be considered as part of a prevention programme in pre-season training.

## 1. Introduction

Pelvic floor dysfunction (PFD) refers to a broad combination of symptoms and anatomical changes associated with abnormal function of the pelvic floor muscles (PFMs) [1] including pelvic organ prolapse (POP) and urinary and fecal incontinence, among others. PFD involves a change in the usual functioning of PFMs and can be categorized as dysfunction caused by elevated PFM tone, PFM pain, reduced PFM tone, or abnormalities in PFM coordination [2].

PFD can be associated with POP, defined as the movement of organs such as the bladder, uterus, and small intestine into or out of the vagina, and urinary and fecal incontinence, namely the involuntary leakage of biological material [3].

Among the types of PFD, urinary incontinence (UI) is the most frequent PFD, impacting 25% of women at some point in their lives [4]. In particular, in females, UI is a common condition whose prevalence can vary between 12.8% and 46.0% [5,6]. Various studies have shown that the risk of incontinence in women between 40–50 years of age within 3 years is 8%, while for those over 65 years old, age is a significant risk factor with a reported rate of about 28%. Even though incontinence becomes more common as people get older, it is incorrect to consider UI as a normal condition of aging. Nevertheless, UI can also occur at a younger age [7,8].

A wide variety of conditions are attributed to UI, including stress UI (SUI), which occurs when urinary incontinence happens involuntarily during activities that raise abdominal pressure, like coughing, sneezing, and exercising; urgency UI occurs when there is a sudden and strong need to urinate before reaching the toilet; mixed UI occurs when both stress and urgency types are present simultaneously; and finally, overflow UI, which is characterized by the involuntary release of extra urine into the bladder, resulting in leakage without feeling the need to urinate even when the bladder is full [9].

The diagnosis of UI is provided by various detection methods: cystometry, emg test, pelvic ultrasound, post-void residual application, urinalysis, and cystoscopy [9].

However, the first step is represented by an accurate anamnesis of the symptoms and clinical examination to verify the presence of clinical signs and risk factors.

Even if the causes of pelvic floor disorder are not properly understood, more than one risk factor for UI has been discussed, however, such as lifestyle habits [10], surgical or obstetrical trauma [11], sexual abuse [12], neuromuscular degenerative diseases or spinal nerve injuries, urinary tract infections, and urological, gynecological, or colorectal diseases [7,13].

Moreover, engaging in intense physical workouts and high-impact exercises has been identified as a relevant risk factor for developing UI [14]. UI prevalence rates range from 10.9% in low-impact sports, such as cycling, to 80% in high-impact sports such as trampoline gymnastics [15].

In detail, high-intensity physical activity is linked to higher intra-abdominal pressure (IAP), which can cause alterations in the morphology and function of the myotendinous structures and connective tissues in the pelvic floor. This could clarify why young nulliparous athletes have UI even without other risk factors [15,16].

UI is particularly relevant in female athletes because it can lead to a feeling of self-consciousness and unease, hindering their participation in sports and impacting their overall well-being and health-related quality of life (HRQoL) [17].

The reluctance of women to talk about their condition results in UI being frequently overlooked and not properly addressed by professionals [18,19]. Moreover, strategies to avoid UI are not commonly incorporated into sports training, and athletes experiencing UI symptoms often employ tactics like wearing absorbent pads, modifying their fluid intake, adjusting their athletic technique, or switching sports to lessen the impact of this condition [20].

Regarding the prevention of UI symptoms in PFD, several studies have shown that pelvic floor muscle training (PFMT) both during pregnancy and after delivery can prevent and treat this condition [21,22]. However, there is still a lack of knowledge on UI prevention in the athletic population [14,23]. The treatment of UI includes conservative therapies, through PFMT, pharmacological treatment, and in exceptional cases surgical treatment [9,24]. Pharmacological and surgical treatments are used in advanced forms when conservative methods fail [24].

To date, conservative management of UI thus represents the first choice because of its effectiveness and safety [24,25].

First of all, it is important to educate athletes on lifestyle modifications, such as changes in diet (avoiding caffeine, alcohol, concentrated sugar, and spicy and acidic food) and gradual weight reduction if overweight [1]. Moreover, PFMT is of fundamental importance to strengthen the pelvic floor. In particular, PFMT includes Kegel exercises, voluntary exercises of contraction and relaxation of PFMs [24,26]. As recommended by NICE (National Institute for Health and Care Excellence) guidelines, the rehabilitation protocol usually consists of a three-month program, supervised by a physical therapist [27].

Moreover, techniques based on electromyographic biofeedback (BFB), electrical stimulation, or weighted vaginal cones have shown interesting results. Namely, electromyographic BFB relies on the use of a vaginal probe to quantify and display the electrical activity of PFMs during exercise, improving exercise quality and consistency [28,29]. Transvaginal Electrical Stimulation (ES) stimulates the PFMs, enhancing strength, especially in severely compromised muscles [30]. Graded weighted vaginal cones are used to promote the contraction of the PFMs to prevent the cone from slipping out [31].

Furthermore, the hypopressive technique involves an organized series of rhythmic postural and sequential respiratory exercises to reduce IAP [32].

However, the effectiveness of conservative treatments in young female athletes is still unclear; therefore, the aim of the present study is to systematically review the literature on the role of pelvic floor rehabilitation in the treatment of urinary incontinence in young nulliparous female athletes. In particular, the main outcomes of interest are the change in urine leakage, frequency, maximum voluntary contraction (MVC), Pubococcygeus (PC) test, changes in quality-of-life, and in the total score of the International Consultation on Incontinence Questionnaire (ICIQ-UI-SF), after a structured rehabilitation protocol for PFD.

## 2. Materials and Methods

### 2.1. Protocol Design

This systematic review was conducted in accordance with the Preferred Reporting Items for Systematic Reviews and Meta-Analyses (PRISMA) statement [33], and it was previously registered in PROSPERO code CRD42024559990.

### 2.2. Search Strategy

Three authors independently performed a bibliographical search in PubMed, Web of Science (WOS), Scopus, and Cochrane Library to select English-language articles, published from inception to April 2024, following the strategy depicted in Table 1.

### 2.3. Selection of Articles

After removing duplicates, two reviewers independently screened all papers for eligibility. In cases of disagreement, consultation with a third reviewer allowed for consensus. Articles were considered eligible if they agreed with the items defined by the following criteria:

- Participant Population: Young nulliparous female athletes with UI (under 35 years of age who practice any sport, whether amateur or professional, with consistency).
- Intervention: PFMT, BFB, functional electrical stimulation, and Kegel exercises.
- Type of study: RCTs, cohort studies, case–control studies, case series, and case reports reporting data prior to and after treatment.
- Outcomes: Urine leakage, frequency, MVC of the pelvic floor muscles, involuntary urine loss with a pad test, quality of life with a questionnaire, the PC test, and change in the total score of the ICIQ-UI-SF.

### 2.4. Data Extraction

Two reviewers independently extracted data from the included studies using a customized data extraction tool on a Microsoft Excel sheet. In cases of disagreement, consensus was achieved with a third reviewer. We extracted the following data: first author and publication year, nationality, population (number of patients included, age, gender, and PFD symptoms), intervention, comparison (if available), outcome measures, and main findings (as reported in Table 2).

### 2.5. Quality Assessment

The quality of the included studies was independently assessed by two reviewers according to the Newcastle Ottawa Scale (NOS) for observational studies and according to the PEDro scale for experimental RCT studies. In cases of disagreement, a third reviewer was consulted to achieve consensus (Table 3 and Table 4). Regarding the NOS, the domains explored are ‘selection of study groups’ (maximum 4 stars), ‘comparability of groups’ (maximum 2 stars), and ‘ascertainment of exposure/outcome’ (maximum 3 stars). The quality classification of the included studies according to NOS score is low (score of 1–3), moderate (score of 4–6), and high quality (score of 7–9) [40]. Quality scores ranged from 0 (lowest) to 9 stars (highest). Regarding the PEDro scale, the first criterion relates to external validity (generalizability of the study), not included in the final calculation as it is not inherent to quality assessment. Items 2–9 refer to internal validity, whereas items 10–11 provide information on the statistical interpretability of the study’s results. There are two options for each item: each “yes” scores one point and each “no” scores zero points. A maximum of ten points can be achieved [41] (Table 3).

Then, to carry out the clinical review, we examined previous guidelines, research queries, adequate evidence, study quality, synthesis of results, and their correct interpretation [42].

## 3. Results

### 3.1. Evidence Synthesis

**Figure 1:**
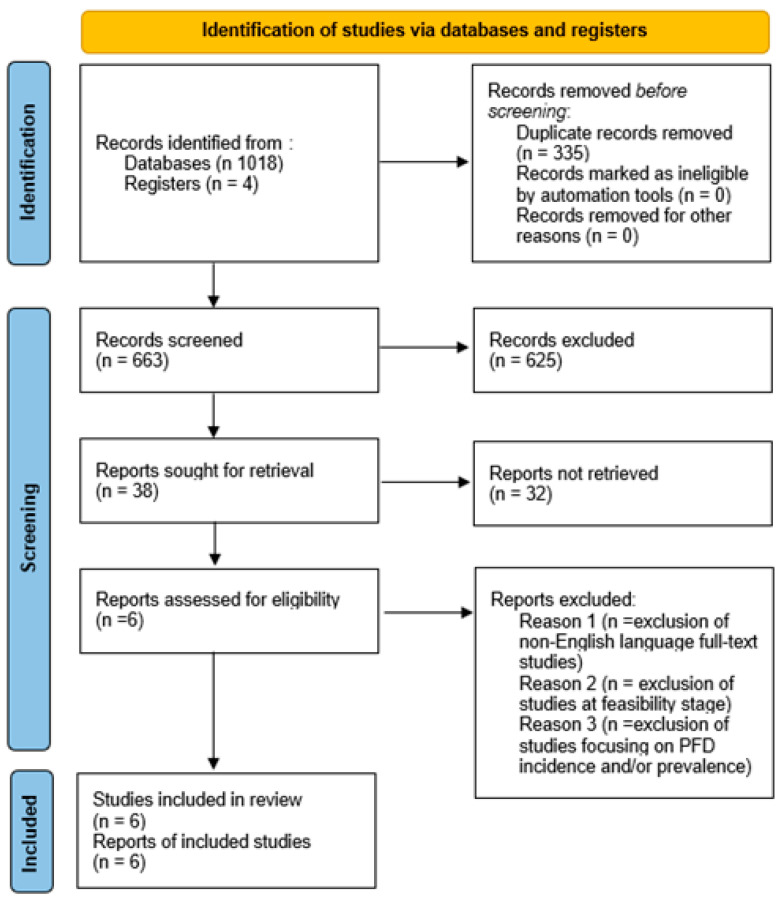
Preferred Reporting Items for Systematic Reviews and Meta-Analyses (PRISMA) flow chart for the systematic literature search and study selection process [33].

A total of 1018 articles were found in all searches of the databases. After removing duplicates, 663 papers were reviewed in terms of title and abstract, and 355 articles were excluded. Thus, we identified 38 full-text articles and retrieved them for a detailed evaluation. Therefore, a total of six studies fulfilled the eligibility criteria and were included in the present review (Figure 1). The included studies were published from April 2010 to April 2024. Two of the selected studies were conducted in the United States (USA [37]; USA and Germany [38]); the other included studies were conducted in Norway [34], Portugal [39], Brazil and Norway [35], Italy [36], and Portugal [39]. 

### 3.2. Synthesis of the Results

A total of 131 female athletes were included, with a mean age of 23.19 years. Table 2 summarizes the main characteristics of the studies included in our systematic review. Three trials were RCTs [34,38,39]. The studies included CrossFit or functional athletes, nulliparous sports students, volleyball athletes, and gymnasts with self-reported UI [34,35,36,37,39]. One trial included both continent and incontinent athletes [38].

### 3.3. Intervention Protocol

#### 3.3.1. Pelvic Floor Muscle Training

Skaug et al. [34] tested the effectiveness of PFMT on SUI in female functional fitness exercisers. Patients were divided into two groups; the interventional group completed a 16-week home-training program and they were given a booklet containing details about PFMT (training position) and demonstration videos about the exercise routine. On the other hand, the control group received only instructions for PFMT by email. The plan included three rounds of 8–12 PFM contractions lasting about 6–8 s each day. They were advised to start their PFMT in a position where they felt able to perform strong PFM contractions, such as lying down or sitting.

Similarly, Ferreira et al. [39] evaluated the effectiveness of PFMT in 32 female volleyball players, divided into two groups. The rehabilitation protocol lasted 3 months and consisted of the awareness and identification of the PFMs, pre-timed PFM contraction prior to occasions of increased IAP pressure, and 30 daily contractions of the pelvic muscles at home. The control group only had access to a pamphlet.

Pires et al. [38] tested the effects of PFMT in fourteen female volleyball athletes with SUI, divided into two groups. The experimental group received a 4-month protocol of PFMT with a daily alert from a physiotherapist regarding the training time. This consisted of three phases: awareness/stabilization, strength training, and power. The awareness phase (2 weeks) aimed to gain awareness of the PFMs, contraction, and relaxation. In the strength phase (2 weeks), the contraction time was greater than the relaxation time, progressively increasing the level of difficulty over time. During the power phase (12 weeks), each athlete had to perform a knack technique (rapid and strong contraction, immediately before and during any increase in downward pressure on the pelvic floor). The athlete’s ability to perform a proper PFM contraction was assessed by the physiotherapist at baseline, through digital palpation. The vaginal resting pressure and then the MVC were evaluated by the same researcher, using manometry.

Da Roza et al. [35] tested the effectiveness of PFMT on UI in sixteen sport students, to whom information material had previously been delivered. However, only seven of them completed the 8-week program and were assessed before and after the intervention period. The intervention program was mediated by three physiotherapists specialized in pelvic floor rehabilitation and it consisted of four stages, conducted for 2 weeks each: (1) becoming familiar with the PFMs through feedback from vaginal palpation, (2) contracting the PFMs in various positions with increasing weights on the legs, (3) practicing PFM contractions while running and walking, and (4) practicing PFM contractions during sports. Furthermore, vaginal palpation was used by the physical therapist to assess the correctness of the voluntary PFM contraction, measured as MVC using a perineometer.

Rodrìguez-Longobardo et al. [37] evaluated the effects of a 12-week Kegel exercise intervention for lower urinary tract symptoms (LUTSs) and UI in nineteen gymnasts with LUTSs. The study protocol was divided into three parts: filling in the baseline ICIQ questionnaire, familiarization with Kegel exercises, and a specific Kegel exercise program. This was led by coaches who received initial training through a theoretical class. This was followed by three 20 min practice sessions where the coaches were taught how to effectively engage the muscles.

#### 3.3.2. Combined Treatment

Rivalta et al. [36] evaluated three female nulliparous agonistic volleyball athletes affected by UI who fulfilled a 48 h voiding diary and underwent urodynamic evaluation. The steps followed were functional electric stimulation (FES) for 20 min once a week for a period of 3 months, by using biphasic intermittent current; BFB for 15 min, once a week, for a period of 3 months; PFMT (300 contractions of the PFMs per day, split into six sessions, switching between isotonic and isometric workouts); and the use of vaginal cones at home (three plastic cones with an inner metal component, alike in shape and size and different in weight).

### 3.4. Outcome Measures

The main primary outcome was a change in the total score of the ICIQ-UI-SF [34,35]. The ICIQ-UI-SF consists of four items: (1) frequency of UI, (2) amount of leakage, and (3) overall impact of UI. From the sum of these three items, the total ICIQ-UI-SF score (between 0 and 21) was calculated. A fourth item included eight questions related to symptoms determining the type of UI. Activity level was measured using the International Physical Activity Questionnaire—Short Form (IPAQ-SF) [35]. Vaginal palpation was used to assess the correctness of the voluntary PFM contraction, and PFM strength was measured as the MVC using a perineometer [35,36,38]. The amount of urinary leakage was assessed using a pad test based on measuring the weight gain of absorbent pads over a period of testing. This is a non-invasive, inexpensive, and objective method [38]. The pad test was modified according to the duration and training program of the athletes (during 2 h [38] or 15 min [39] of physical activity). According to Ferreira et al., athletes were considered incontinent when urinary loss exceeds one gram [39]. The frequency of UI episodes consisted of a daily log of urinary leakage for seven consecutive days [38] or a 48 h voiding diary [36]. King’s Health Questionnaire was used to assess quality of life [38]. The PC test was also used to document PFM function and strength and was graded based on the Modified Oxford Grading Scale [36]. A Self-Efficacy Scale was used by Skaug et al. to evaluate changes in symptoms of SUI, and the participants were asked to rate their perceived change in SUI with a validated 7-point scale (Patient Global Impression (PGI)) with response choices ranging from ‘very much better’ to ‘very much worse’ [34].

#### 3.4.1. Urinary Incontinence

##### PFMT

Skaug et al. reported, after a 16-week program, a mean difference between the two groups of −1.4 (95% CI: −2.6 to −0.2) in the change in ICIQ-UI-SF score in favor of the PFMT group. The PFMT group completed a mean of 70% (SD: 23) of the prescribed protocol. Moreover, 64% in the PFMT group versus 8% in the control group reported improved symptoms of SUI (*p* < 0.001, RR:7.96, 95% CI 2.03 to 31.19) on the PGI-I scale. No adverse effects were reported. There were no differences in the change in PFM resting pressure, strength, or endurance between the groups [34].

Pires et al. proved that the experimental group showed improved MVC (*p* < 0.001) and reduced urine loss (*p* = 0.025), with significant differences between the groups. The percentage of urine loss decreased in the experimental group, from 71.4 to 42.9%, suggesting that the protocol intervention for 16 weeks may help athletes with SUI [38].

Ferreira et al. reported that the amount of urine leakage decreased in 45.5% of athletes under PFMRP intervention and in 4.9% of athletes in the CG, with statistical differences between the groups (*p* < 0.001). The reduction in the frequency of urinary leakage was 14.3% in the EG and 0.05% in the CG, a statistically significant difference between the groups (*p* < 0.001) [39].

Da Roza et al. showed that participants in the program had a significantly greater frequency of leakage (1.6 ± 1.5 vs. 1.0 ± 0.0) and a larger impact on their QOL (2.9 ± 3.8 vs. 0.8 ± 0.9) compared with the sports students that dropped out of the exercise program (*p* = 0.016 and *p* = 0.007, respectively). Vaginal resting pressure improved by 17.4 cmH_2_O (SD 6.7), *p* = 0.04, and MVC improved by 16.4 cmH_2_O (SD 5.8), *p* = 0.04. ICIQ-UI-SF score, frequency, and amount of leakage showed statistically significant improvement after an 8-week PFMT program [35]. However, Rodrìguez-Longobardo et al. did not find any significant differences in LUTSs and quality of life variables after the exercise intervention (*p* > 0.05) [37].

##### Combined Treatment

Rivalta et al. reported that after the combined rehabilitation program, none of the athletes reported UI requiring a device (pad or panty liner use) nor did they refer to urine leakage during sport and fitness activities or during daily life. The PC test improved in all of the athletes. No side effects or complications connected to the complete PFR were recorded [36].

#### 3.4.2. Limitations

Many studies report a small sample size as a limitation of the trial [35,37,38]. One of the studies reported as a limit of their research the high dropout rate because of the nature of the training program, which was time consuming, and the need for specific equipment [35]. An additional limitation was a non-randomized design [35,36]. Rodriguez-Longobardo et al. reported the short duration of the study and the use of self-reported data instead of objective measurements as limitations [37]. Furthermore, objective measurements normally imply the use of intracavitary devices, but some of the participants were young and had not reached menarche yet, and this led to this type of practice not being used [37]. In addition, self-reported questionnaires can be affected by recall bias and the treatment effect may have been underestimated by categorical responses [34]. A limitation of Skaug et al.’s study is the lack of supervised training and follow-up assessments, which may have negatively influenced adherence, the intensity of the program, and the frequency of UI [34]. Moreover, the authors reported that the questionnaire did not include questions regarding urinary leakage during functional-specific exercises, so any improvements in UI during these exercises may not have been considered by the ICIQ-UI-SF [34]. Questions regarding the use of tampons or other anti-incontinence devices were also not included, and these devices may decrease the amount of urine leakage. Finally, in this study, the results may have been influenced by response bias due to missing outcome data from four participants at post-test [34].

### 3.5. Quality Score

Three of the six studies considered (50%) were of excellent quality, as shown in Table 3, and three were of moderate quality (50%), as shown in Table 4.

## 4. Discussion

High-impact sports athletes, e.g., those involved in gymnastics or trampoline jumping, showed a higher prevalence of SUI [43,44,45,46,47,48]. However, this topic is still understudied, due to the reluctance of young athletes to talk about their symptoms and limited knowledge regarding pelvic floor rehabilitation in female athletes. Therefore, the purpose of this review was to evaluate the effectiveness of available conservative treatments in female athletes in an at-risk group for UI. Considering the results of the included studies, PFMT is the most common proposed treatment and resulted in an effective strategy for the prevention and management of UI. Specifically, PFMT has shown interesting results in improving MVC [35,37,38], which is an electromyographic index, useful for analyzing PFM strength in counteracting IAP-positive variation during physical activity [49,50]. Furthermore, PFMT has a significant effect on the reduction in urine leakage, as shown after a 4-month PFMT program proposed by Pires et al. [38], 4-month combined treatment by Rivalta et al. [36], and a 3-month PFMT program proposed by Ferreira et al. [39], as measured using pad tests or self-reported in the ICIQ-UI-SF [34,35,39]. In addition, the effect of PFMT on quality of life was evident, after two to four months of treatment, with a positive influence on sports motivation. This agrees with the research by Radzimińska et al. [51], which shows that PFMT significantly improves the quality of life of women with UI, as an important determinant of their physical, mental, and social functioning. However, Rodriguez-Longobardo et al. [37] did not reach the same conclusions with a Kegel exercise program. In their study, the authors observed no reductions in symptoms in the study group and they even intensified after 12 weeks. According to the authors, this could be due to the underage gymnasts, and a general lack of awareness of the topic may have affected the results. In fact, it was found that treatment success also depends on correct identification of the muscles involved, awareness, and protocol adherence to exercise. Correct patient education can thus avoid the incorrect and concomitant contraction of other muscles such as the rectus abdominis, the adductor of the thigh, and the gluteus maximus, which greatly decreases the contractile activity of the PFMs [38]. In this regard, the results of the present review highlight that the combination of PFMT with an educational program act in synergy in reducing UI symptoms in female athletes. The educational program could play a role in improving awareness of the problem, which represents the first step for correct identification and treatment [52]. It can be carried out through practical explanations by a specialized team with the aid of information material, e.g., brochures and videos [53,54]. Moreover, it is essential that exercise is supervised. In the studies included in this review, female athletes executed a specific training program supervised by a physical therapist. In high-impact sports, there is a need for specialized professionals, with specific education on PFD, to raise the awareness of athletes and target the appropriate skills for UI. This is in accordance with NICE guidelines [55], which highlight the role of the therapist in the assessment of the woman’s ability to perform effective PFM contractions and relaxations and, consequently, drawing up a customized training program. Furthermore, PFMT can be combined with other interventions, as proposed by Rivalta et al. [36], evaluating combined treatment by using PFMT and BFB, FES, and vaginal cones, achieving good results in reducing the frequency of urine leakage. In conclusion, conservative treatment with a personalized PMFT program alone is proven to be an effective treatment for UI, strengthening and enhancing the support of pelvic floor structures to counteract ground reaction forces and the increased IAP during training and competition, which can lead to the development of UI [56], especially in high-impact sports. In addition, considering that high-impact sports are one of the triggers in the development of UI in female athletes, also according to Culleton-Quinn et al. [57], it would be appropriate to include a PFMT program in preseason training. Despite the limited number of clinical studies on interventions for UI in female athletes, they almost unanimously agree that PFMT represents a simple and safe treatment for UI, with few side effects reported during the treatment period.

### Study Limitations 

This systematic review is limited by the small number of studies available. Despite the extensive search strategy, it is possible that some studies were not correctly incorporated. In addition, the samples examined in each included study were small themselves [37]. Furthermore, it was not possible to conduct a meta-analysis because of the heterogeneous nature of the included studies, such as the lack of control groups in the three studies included. It is also believed that symptoms such as urine leakage are still a taboo topic, and many athletes try to hide it from doctors and healthcare providers, mistakenly thinking of it as a natural occurrence and not a relevant problem. Another limitation is the lack of studies on the male population, although the literature shows a correlation between LUTSs and years of high-intensity cycling [58]. It is possible, however, that male athletes may also benefit from PFMT. Therefore, the usefulness of studying this cluster to evaluate the inclusion of PFMT as an integral part of training for male athletes also becomes apparent.

## 5. Conclusions

In conclusion, the results of this study show that PMFT represents an effective treatment for both the prevention and management of UI. Therefore, PFTM could be considered a viable, safe, and cost-effective treatment option to be included as a training program in young female athletes, especially in high-impact sports, to prevent the occurrence of urinary tract symptoms. A customized rehabilitation program allows female athletes to acquire strategies for proper management of IAP during competitions and in daily life. Furthermore, it would be appropriate to integrate an educational program with the aim of improving the perception of UI-related symptoms and knowledge of the various therapeutic solutions.

## Figures and Tables

**Table 1:** Search strategy.

PubMed (pelvic floor dysfunction) OR (urinary incontinence) OR (dyspareunia) OR (dysuria)) AND ((sport) OR (sports)) AND ((female) OR (woman) OR (women) OR (girl)) AND ((rehabilitation) OR (rehab) OR (pelvic rehabilitation) OR (exercise))
Scopus (pelvic floor dysfunction) OR (urinary incontinence) OR (dyspareunia) OR (dysuria)) AND ((sport) OR (sports)) AND ((female) OR (woman) OR (women) OR (girl)) AND ((rehabilitation) OR (rehab) OR (pelvic rehabilitation) OR (exercise))
Web of Science (pelvic floor dysfunction) OR (urinary incontinence) OR (dyspareunia) OR (dysuria)) AND ((sport) OR (sports)) AND ((female) OR (woman) OR (women) OR (girl)) AND ((rehabilitation) OR (rehab) OR (pelvic rehabilitation) OR (exercise))
Cochrane Library (pelvic floor dysfunction) OR (urinary incontinence) OR (dyspareunia) OR (dysuria)) AND ((sport) OR (sports)) AND ((female) OR (woman) OR (women) OR (girl)) AND ((rehabilitation) OR (rehab) OR (pelvic rehabilitation) OR (exercise))

**Table 2:** Main characteristics of studies included in the present systematic review.

Article	Nationality	Type of Study	Study Group	Control Group	Intervention	Comparison	Outcome Measure and Time-Point Assessments	Main Findings
Skaug et al. *Br J Sports Med* 2024 [34]	Norway	RCT	*n* = 22; 22F; mean age: 33.5; CrossFit or functional fitness training with stress urinary incontinence (SUI)	*n* = 25; 25F; mean age: 33.5; CrossFit or functional fitness training with SUI	PFMT 16-week program	No education on PFMT, lifestyle modifications, or other pelvic floor treatment options	Change in the total score of the ICIQ-UI-SF at baseline and at the end of 16 weeks of rehabilitative treatment	Mean difference between groups of −1.4 (95% CI: −2.6 to −0.2) in the change in ICIQ-UI-SF score in favor of the PFMT group; 64% in the PFMT group versus 8% in the control group reported improved symptoms of SUI (*p* < 0.001, relative risk: 7.96, 95% CI, 2.03 to 31.19)
Da Roza et al. *Int Urogynecol J* 2012 [35]	Portugal, Brazil, and Norway	Pilot study	*n* = 16; 16F; mean age: 20.0 ± 0.8 years, only 7 of them completed the 8-week program, nulliparous sport students with UI	None	PFMT 8-week program	None	Change in the total score of the ICIQ-UI-SF and PFM strength at baseline and at the end of 8 weeks program	Vaginal resting pressure improved by 17.4 cmH_2_O (SD 6.7), *p* = 0.04, and MVC by 16.4 cmH_2_O (SD 5.8), *p* = 0.04. ICIQ-UI-SF score and frequency and amount of leakage showed statistically significant improvement
Rivalta et al. *Health Care Women Int.* 2010 [36]	Italy	Observational studies	*n* = 3; 3F; mean age: 30.6 years, volleyball athletes affected by UI		BFB, functional electrical stimulation, PFMT, and vaginal cones		UI defined as the need for a pad or panty liner during sport or daily life at baseline and at the end of a 4-month rehabilitation program with urogynecological evaluation and a 48 h voiding diary and changes in PC test	Patients reported pad or panty liner usage of 1–2 per day at baseline. After the combined rehabilitation program, none of them reported UI requiring devices (pad or panty liner use), and the PC test improved in all of the athletes
Rodriguez-Longobardo et al. *Urogynecology* (Phila) 2023 [37]	USA	Prospective Cohort Study	*n* = 19; 19F; mean age of 13.21/−1.84; gymnasts with LUTSs (Lower Urinary Tract Symptoms)	None	Kegel exercises—12-week program	None	Change in the total score of the International Consultation on Incontinence Questionnaire—Female LUTS validated questionnaire at baseline and at the end of the 12-week intervention	No significant differences in LUTSs and quality of life variables were observed after the exercise intervention (*p* > 0.05)
Pires et al. *International Journal of Sports Medicine* 2020 [38]	USA and Germany	RCT	*n* = 7, 7F; mean age: 22.6 years; athletes, both continent and incontinent	*n* = 7, 7F; aged between 18 and 30 years; athletes, both continent and incontinent	PFMT 4-month program	No education on PFMT, lifestyle modifications, or other pelvic floor treatment options	MVC was evaluated with a perineometer, involuntary urine loss was evaluated with a pad test, and quality of life was evaluated with the King’s Health Questionnaire at baseline and at the end of the 4-month program	The experimental group improved MVC (*p* < 0.001) and reduced urine loss (*p* = 0.025). The percentage of urine loss decreased in the experimental group, from 71.4 to 42.9%
Ferreira et al. *Revista da Associacao Medica Brasileira* 2014 [39]	Portugal	RCT	*n* = 16; 16F; mean age: 19.4 years, female volleyball athletes with urinary leakage	*n* = 16; 16F; mean age: 19.1 years; female volleyball athletes with urinary leakage	PFMTRP 3-month program	No education on PFMT, lifestyle modifications, or other pelvic floor treatment options	Questionnaires, the pad test (amount of urinary leakage), and frequency record of urinary leakage (7-day diary) before and after 3 months of PFMRP	The amount of urine leakage decreased in 45.5% of athletes under PFMRP intervention and in 4.9% of athletes in the CG (*p* < 0.001). The reduction in the frequency of urinary leakage was 14.3% in the EG and 0.05% in the CG (*p* < 0.001)

Abbreviations: RCT (Randomized Control Trial); SUI (Stress Urinary Incontinence); biofeedback BFB PMFT (Pelvic Floor Muscle Training); ICIQ-UI-SF (International Consultation on Incontinence Questionnaire—Short Form); CI (Confidence Interval); UI (Urinary incontinence); MVC (Maximum Voluntary Contraction); PC test (Pubococcygeus test); LUTSs (Lower Urinary Tract Symptoms); PFMTRP (Pelvic Floor Muscle Training Rehabilitation Program); CG (Control Group).

**Table 3:** PEDro scale for the methodological quality assessment of experimental RCT studies.

Criteria for the Quality Scoring												
Articles	1	2	3	4	5	6	7	8	9	10	11	Risk of Bias
Skaug et al. *Br J Sports Med* 2024 [34]	11	11	11	11	00	00	00	11	11	11	11	Low risk
Pires et al. *International Journal of Sports Medicine* 2020 [38]	11	11	11	11	11	00	11	11	11	11	11	Low risk
Ferreira et al. *Revista da Associacao Medica Brasileira* 2014 [39]	11	11	11	11	00	00	00	11	11	11	11	Low risk

**Table 4:** Newcastle Ottawa Scale (NOS) score for the methodological quality assessment of observational studies.

Study	S1	S2	S3	S4	C	E1	E2	E3	Total Scale	Quality
Da Roza et al. *Int Urogynecol J* 2012 [35]	*	*	–	–	*	–	*	–	4	Moderate
Rivalta et al. *Health Care Women Int.* 2010 [36]	*	*	–	–	*	–	*	–	4	Moderate
Rodriguez-Longobarto et al. *Urogynecology* (Phila) 2023 [37]	*	*	–	–	*	–	*	–	4	Moderate

## Data Availability

Data available on request.
